# Drivers and Spatio-Temporal Extent of Hyporheic Patch Variation: Implications for Sampling

**DOI:** 10.1371/journal.pone.0042046

**Published:** 2012-07-30

**Authors:** Alexander Braun, Karl Auerswald, Juergen Geist

**Affiliations:** 1 Aquatic Systems Biology Unit, Department of Ecology and Ecosystem Management, Technische Universität München, Freising, Germany; 2 Lehrstuhl für Grünlandlehre, Department of Plant Science, Technische Universität München, Freising, Germany; Utrecht University, Netherlands

## Abstract

The hyporheic zone in stream ecosystems is a heterogeneous key habitat for species across many taxa. Consequently, it attracts high attention among freshwater scientists, but generally applicable guidelines on sampling strategies are lacking. Thus, the objective of this study was to develop and validate such sampling guidelines. Applying geostatistical analysis, we quantified the spatio-temporal variability of parameters, which characterize the physico-chemical substratum conditions in the hyporheic zone. We investigated eight stream reaches in six small streams that are typical for the majority of temperate areas. Data was collected on two occasions in six stream reaches (development data), and once in two additional reaches, after one year (validation data). In this study, the term spatial variability refers to patch contrast (patch to patch variance) and patch size (spatial extent of a patch). Patch contrast of hyporheic parameters (specific conductance, pH and dissolved oxygen) increased with macrophyte cover (r^2^ = 0.95, p<0.001), while patch size of hyporheic parameters decreased from 6 to 2 m with increasing sinuosity of the stream course (r^2^ = 0.91, p<0.001), irrespective of the time of year. Since the spatial variability of hyporheic parameters varied between stream reaches, our results suggest that sampling design should be adapted to suit specific stream reaches. The distance between sampling sites should be inversely related to the sinuosity, while the number of samples should be related to macrophyte cover.

## Introduction

The hyporheic zone in stream ecosystems is highly heterogeneous. Its biotic and abiotic properties vary spatially and temporally [1], [2]. Many studies have recognized abiotic heterogeneity as a significant driver of biodiversity with effects on genetic diversity [3], population dynamics [4] and species diversity [5]. Also, the hyporheic zone is considered a key habitat for species across many taxa and levels of organization, including microorganisms, periphyton, invertebrates and fishes [6]. Many critically endangered freshwater taxa directly or indirectly depend on the properties of the hyporheic zone for completion of their life cycles [7], [8]. Additionally, it is essential for ecosystem functions related to turnover and retention of nutrients and contaminants. Thus, the hyporheic zone attracts high attention among freshwater scientists; however, despite of recent progress in frameworks for hyporheic sampling [9], [10], [11], generally applicable strategies for sampling are still lacking.

A proper sampling strategy should account for accuracy, precision, representativeness and autocorrelation of the data. To ensure a proper sampling design, critical decisions have to be made for every ecological study in advance. These decisions need to consider both the spatial and temporal variability of the stream reach under study [12], [13]. In particular, decisions related to the spatial variability comprise (i) the stream reach to investigate (larger spatial scale), (ii) the placement of sampling sites - either random sampling or any kind of systematic sampling design - within the stream reach (smaller spatial scale), (iii) the distance between sampling sites, and the (iv) total number of samples; with subdivisions (ii), (iii) and (iv) determining the size of the investigated area. Decisions related to the temporal variability are the (v) time of sampling (regarding different time scales, from a daily scale to an annual scale) and (vi) potential temporal repetitions. Lastly, the researcher has to decide, (vii) whether one sampling design is appropriate for all stream reaches included in a study. Without a scientific framework for hyporheic zone sampling, doubtful decisions are likely to be made, which additionally may vary between researchers, and even between studies with the same researcher [14]. Therefore, this paper addresses how unbiased data collection - in the sense to avoid or correct for spatially autocorrelated samples - may be performed using the example of the hyporheic zone. It is worth noting that spatial autocorrelation needs to be considered in all possible sampling designs and is thus inevitably to be taken into account when designing a sampling strategy.

Geostatistics are highly suitable to analyze spatial patterns, e.g. spatial autocorrelation [15], [16], yet they are hardly considered in aquatic ecology. A geostatistical approach, which quantifies spatial autocorrelation, might therefore provide a step forward in ecological research of the hyporheic zone. Spatial autocorrelation is a measure of the spatial dependence, based on the principle, that nearby sampling sites are more similar than distant sampling sites. Derived conclusions comprise i) the patch contrast, indicating the quantitative difference between two patches and ii) the patch size, representing the spatial extension of a patch, where a patch is herein defined as an area differing from its surroundings [17]. These results will influence recommendations for sampling designs, since the distance between two sampling sites should be related to the specific local patch size [18]. Thereby, we cover an unsolved key issue for environmental monitoring or any assessment of habitat aiming to assess the ecological status in stream ecosystems as e.g. required by the EU Water Framework Directive. A proper sampling design may, however, differ between streams, between parameters to be measured and according to the time of sampling.

The application of geostatistical approaches in ecology and environmental monitoring can be a milestone for two additional reasons. First, determining the drivers of patch contrast and patch size may enhance the understanding of ecosystem functioning and its contribution to biodiversity (e.g. patch richness and the number of ecological niches are positively correlated). Second, spatial dependence of parameters has to be considered to avoid the introduction of a systematic bias which may lead to false interpretations [19]. Autocorrelated and thus spatially dependent parameters must not be judged as independent and should therefore not be used in statistical “standard” tests that assume independence. Ignoring spatial autocorrelation can lead to wrong declaration of significance based on an under-estimation of confidence levels [20].

The objectives of this study were to (i) quantify the spatial variability (patch contrast and patch size) of the hyporheic zone of different stream reaches and its temporal variation, (ii) determine the drivers of spatial variability, and thus (iii) give recommendations how to conduct sampling in the hyporheic zone. For this purpose, data was collected on two occasions on six stream reaches to analyze the spatial variability in the hyporheic zone. The resulting model was verified at two additional stream reaches after one year.

## Results

### Characterization of Investigated Stream Reaches

**Figure 1:**
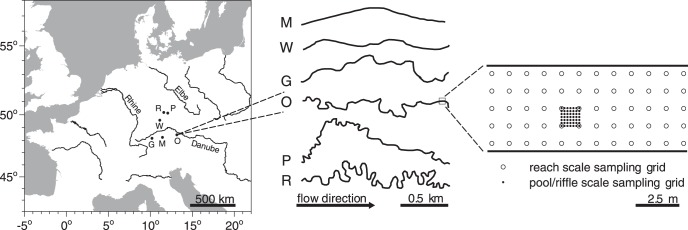
Map and sampling design of the investigated stream reaches. Map of Central Europe comprising the locations of the investigated stream reaches (left panel), their plan view morphologies 2 km upstream of the sampled reach (middle) and a scheme of the nested sampling design (right panel). Open symbols in the right panel denote the reach scale grid (distances of 1 m), closed symbols denote the pool/riffle scale grid (distances of approx. 17 cm). Codes for the stream reaches are M  =  Moosach, W  =  Wiesent, G  =  Guenz, O  =  Grosse Ohe, P  =  Perlenbach, R  =  Suedliche Regnitz.

**Figure 2:**
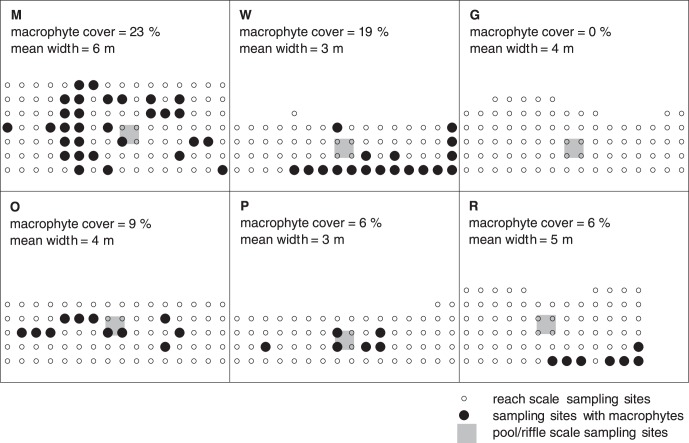
Detailed sampling design and macrophyte cover. The adjusted sampling designs according to stream widths (circles at 1 m distance represent sampling sites of the reach scale grid). Solid circles indicate sampling sites with submerged macrophytes, open circles indicate sampling sites without submerged macrophytes. The area of the pool/riffle scale grid is denoted in grey (the 49 sampling sites of the pool/riffle scale grid are not explicitly shown here, but see Fig. 1). The stream reaches are M  =  Moosach, W  =  Wiesent, G  =  Guenz, O  =  Grosse Ohe, P  =  Perlenbach, R  =  Suedliche Regnitz.

The stream reaches (Fig. 1) comprised a wide variety of conditions within the given focus of small streams, which are typical for the majority of temperate regions. Stream reaches with calcareous bedrock geology (M, W, G) had significantly (p<0.001) greater mean values for specific conductance and pH than reaches with siliceous bedrock geology (O, R, P, Table 1). Additionally, each stream reach was significantly (p<0.01) different to all other stream reaches for specific conductance and pH. At least three parameters at each stream reach were clearly different between sampling dates. Two of the stream reaches (M, W) had a high macrophyte cover (higher than 15%), three (O, R, P) had a low cover (lower than 10%) and one (G) had no macrophytes. Where present, macrophytes formed several patches within the investigated reaches (Fig. 2). Their locations and frequencies remained constant from June to September, though an increase in plant biomass was apparent. Comparing the streams’ sinuosities deduced from the stream courses 1.5 km upstream of the investigated stream reaches, M was minor meandering, almost representing a straightened channel (sinuosity: 0.06), whereas the streams from W (0.2) to G (0.3), O (0.6), P (0.7) and R (1.0) had increasing values of meandering.

**Table 1:** Physico-chemical properties of the study sites.

Stream	pH		Specificconductance		Dissolvedoxygen		Depth		Current atsurface		Current atsubstratum	
			(µS cm^−1^)		(mg L^−1^)		(cm)		(m s^−1^)		(m s^−1^)	
M	7.6^b^	±0.2	793^b^	±57	5.2^b^	±3.3	55^b^	±16	0.20^b^	±0.11	0.12^b^	±0.10
W	7.7^c^	±0.2	762^c^	±61	6.1^a^	±3.4	38^c^	±15	0.28^ad^	±0.25	0.19^ac^	±0.18
G	7.8^a^	±0.2	608^a^	±22	6.0^a^	±1.7	28^a^	±24	0.31^a^	±0.20	0.19^ac^	±0.14
O	6.4^d^	±0.2	141^d^	±30	5.8^a^	±2.0	41^c^	±12	0.27^cd^	±0.17	0.21^c^	±0.16
P	7.1^e^	±0.1	255^e^	±17	4.5^c^	±2.3	27^a^	±16	0.24^c^	±0.15	0.17^ad^	±0.16
R	7.4^f^	±0.1	327^f^	±113	3.8^d^	±2.6	39^c^	±14	0.25^cd^	±0.15	0.15^bd^	±0.13

Means and standard deviations of measured parameters to characterize streams Moosach (M), Wiesent (W), Guenz (G), Grosse Ohe (O), Perlenbach (P) and Suedliche Regnitz (R). Mean values followed by the same letter within a column are not significantly different. The parameters indicated as current represent the current speed measured either 5 cm below the water surface (“current at surface”) or 5 cm above substratum (“current at substratum”).

Within stream reaches, sampling sites with submerged macrophytes had significantly (p<0.01) higher mean values of specific conductance and significantly (p<0.001) lower mean values of dissolved oxygen and pH. The tests were only performed for M and W. For the reaches O, R, P and G testing was not possible, because the numbers of sampling sites with submerged macrophytes were too low. Furthermore, surface current speed was significantly (p<0.01) higher at sampling sites with macrophytes (e.g., for stream M surface current speed was on average 0.23 m s^−1^ at sampling sites with macrophytes and 0.17 m s^−1^ at sites without). This can be expected from the Venturi effect caused by the narrowing of the flow cross-sectional area by the macrophytes. In contrast, the current speed above the substratum within one reach (bare sites *vs* sites with macrophytes) was significantly (p<0.01) slower at sampling sites with macrophytes due to the increase in hydraulic roughness within the macrophyte assemblages (e.g. for stream M, the current speed above the substratum was on average 0.11 m s^−1^ at sampling sites with macrophytes and 0.16 m s^−1^ at sites without).

### Analysis of Spatial Variability

Stream ecosystems are highly heterogeneous regarding abiotic properties. Here, the range (maximum – minimum value) for each parameter within a stream reach was of similar amplitude as the range among the stream reaches’ averages. For example, the range in pH was 1.54 within the stream reach O, while it was 1.58 between all stream reaches, despite the fact that the streams drained siliceous and calcareous catchments, respectively.

**Figure 3:**
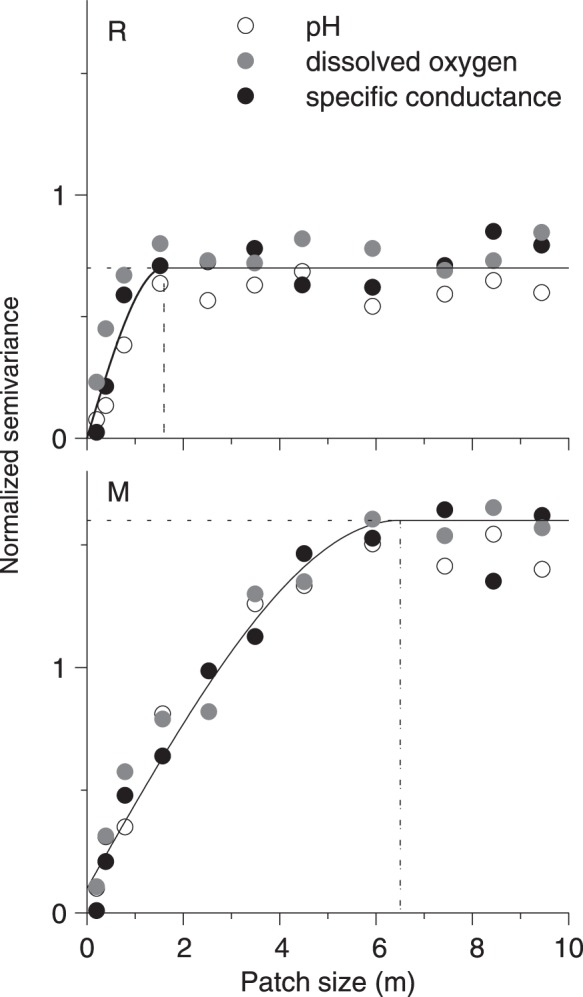
Geostatistical analysis: variograms of contrasting stream reaches. Normalized semivariances of the hyporheic parameters specific conductance, dissolved oxygen and pH as a function of distance between sampling sites for two contrasting stream reaches (R and M). A spherical variogram model (solid line is only for illustration; for analysis, models were fitted individually for each parameter) quantifies the geostatistical parameters sill (here denoted patch contrast and representing the maximum value, horizontal dashed line) and range (here denoted patch size and representing the distance at which the patch contrast is reached, vertical dashed line). Markers show the mean of both sampling dates from the development data set. R = Suedliche Regnitz, M  =  Moosach.

**Figure 4:**
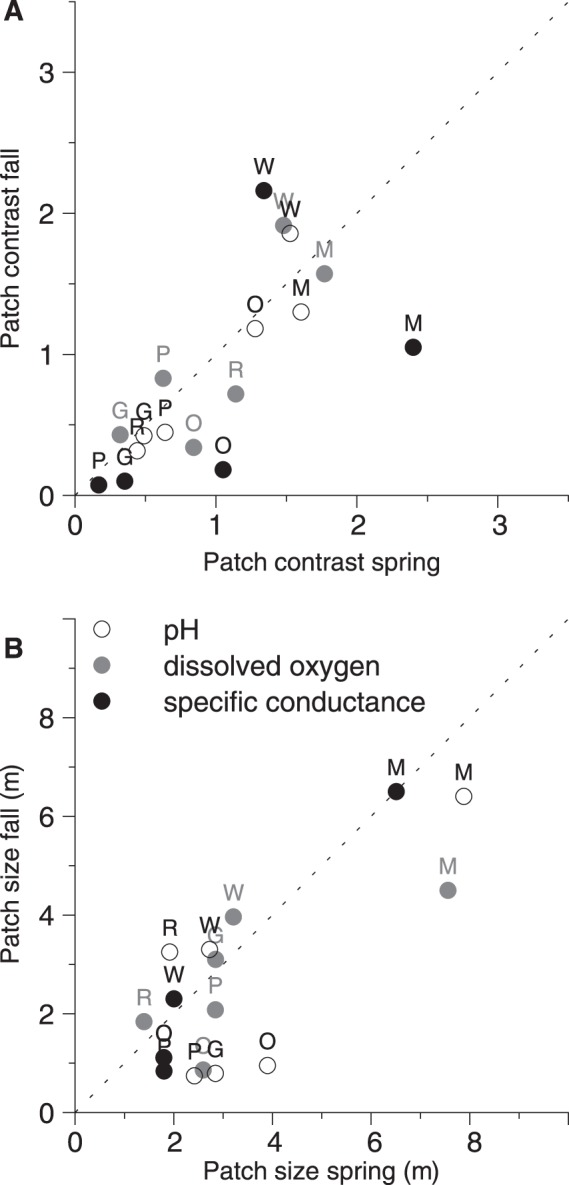
Results of geostatistical analysis: Patch contrasts and patch sizes. Results of the geostatistical analysis of all stream reaches at both dates of the development data: patch contrasts (A) and patch sizes (B) of hyporheic parameters. Dashed lines (y  =  x) indicate no temporal variation. Patch contrast is shown as normalized value, where 1 represents the mean for each parameter. Markers are coded with a capital letter for the stream reach. M  =  Moosach (without validation data), W  =  Wiesent, G  =  Guenz, O  =  Grosse Ohe, P  =  Perlenbach, R  =  Suedliche Regnitz.

In all stream reaches at both dates, all parameters were spatially autocorrelated with one exception (specific conductance in G did not exhibit spatial autocorrelation at both sampling dates). This is illustrated for the hyporheic parameters of two contrasting streams in Fig. 3. The patch sizes (range of autocorrelation) varied between 1 m and 15 m. After normalization, all hyporheic parameters within stream reaches revealed essentially identical patterns regarding autocorrelation (Fig. 3). Though, sampling dates differed in their autocorrelation at stream reach M, there was no consistent difference in autocorrelation between sampling dates over all stream reaches, despite the change of absolute values (Fig. 4). For investigated scales (pool/riffle and reach), no patterns of hierarchical patch structures were detected, which would have been evident as nested variograms.

Considering the hyporheic parameters, stream reaches with different bedrock geology (M, W, G *vs* O, R, P) were not significantly different in patch contrast and patch size. However, stream reaches M and W had significantly (p<0.01) higher patch contrasts (by about factor 2) compared to G, O, P and R (Fig. 4 A). M had significantly (p<0.001) larger patch sizes (approx. 6.5 m) compared to all other stream reaches and W had significantly (p<0.05) larger patch sizes (approx. 3 m) compared to O, P and R (approx. 2 m, Fig. 4 B).

Regarding flow parameters, patch contrasts and patch sizes (mean 4.8 m) showed neither a significant difference between geochemistry units, nor between stream reaches, nor a clear difference between sampling dates and any other grouping. Hence, spatial variability of flow parameters is relatively uniform among the given range of stream conditions. It has to be noted that sampling was carried out during base flow conditions as it was not intended to capture peak flow or low water conditions.

### Factors Affecting the Spatial Variability

**Figure 5:**
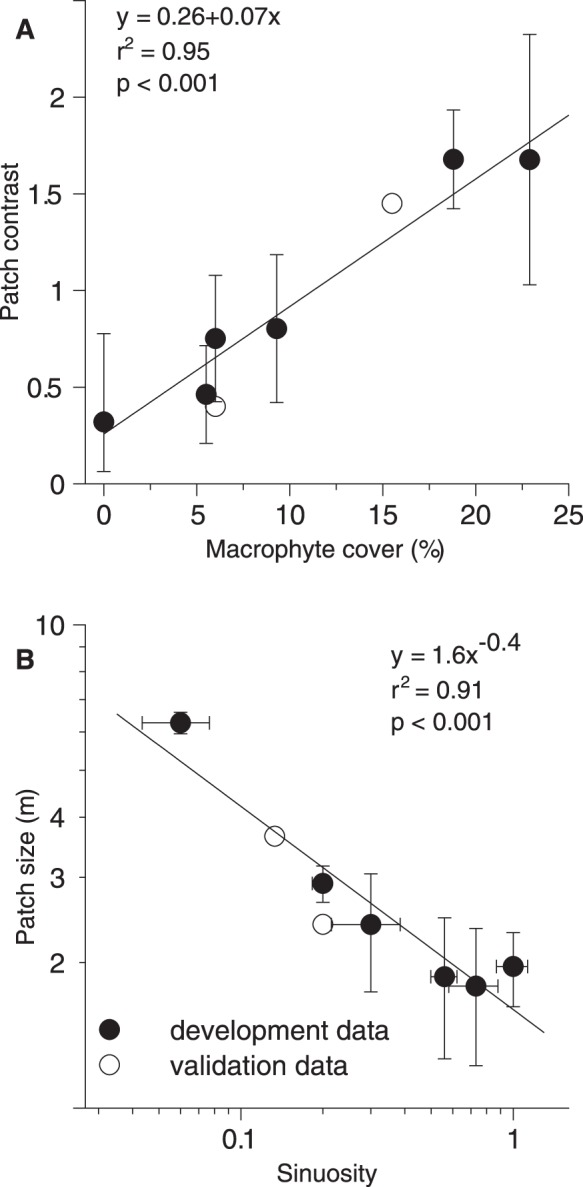
Models to predict patch contrast and patch size. Correlations of normalized patch contrast of hyporheic parameters with the submerged macrophyte cover (A) and correlation of mean patch size of hyporheic parameters with the sinuosity for a 1.5 km long stretch upstream of the sampling site (B). Note the logarithmic scale for (B). Means of patch contrasts and patch sizes were calculated for each stream over both sampling dates and three (pH, specific conductance, dissolved oxygen) parameters (n = 6). Vertical bars show the respective standard deviation. Horizontal bars in (B) display the range in sinuosity for 0.5 to 2 km long stretches. Closed circles denote the development data, open circles denote the validation data. Statistics have been calculated without validation data.


The following parameters were tested as potential drivers for the spatial variability (patch contrast and patch size) but showed no significant correlation: mean width and width variance, mean current speed and current speed variance (both currents, 5 cm below the water surface and 5 cm above the streambed), mean depth and depth variance. Instead, the spatial variability in the hyporheic zone was affected by two other factors, biotic and abiotic. The biotic factor was the macrophyte cover, which significantly (p<0.001) increased patch contrasts of hyporheic parameters as indicated by the linear regression model (p<0.001, r^2^ = 0.95, Fig. 5 A).

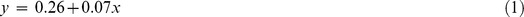


with *y* representing the dimensionless patch contrast and *x* representing the macrophyte cover. The linear regression model was suggested by the AICc as the optimum model. The regression shows that the patch contrasts (and thus the variance) increased by more than factor 2 if the macrophyte cover increased from 5 to 15%. This was apparently true for all hyporheic parameters, which did not differ clearly in this respect.

Sinuosity upstream of the sampling sites strongly influenced the spatial variability in the hyporheic zone. Correlations were significant between the sinuosity for linear distances of 0.5 km (r^2^ = 0.67), 1 km (r^2^ = 0.82) and 1.5 km (r^2^ = 0.86) and the patch sizes, whereas the sinuosities calculated for 0.2 km (r^2^ = 0.17) and 2 km (r^2^ = 0.25) linear distances did not correlate significantly. The model of the linear distance with the strongest correlation (sinuosity of the stream course 1.5 km upstream of the investigated stream reach) was (p<0.001, r^2^ = 0.86, Fig. 5 B)


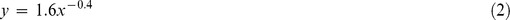


with *y* representing the patch size and *x* representing the sinuosity. The power function was suggested by the AICc as the optimum model. Eqn 2 indicates that the patch sizes decreased with increasing sinuosity. Remarkably, the rather small difference in sinuosity between W and M caused the patch size to increase more than twofold (from 3 to 6 m), whereas the rather big difference in sinuosity between W and O, P and R caused only a minor decrease in patch size (from 3 to 2 m). The sinuosity also correlated closely (r^2^ = 0.975) with the Reynolds number, as calculated from kinematic viscosity, annual discharge, wetted perimeter and flow cross-sectional area as given by the data in Tables 1, 2 and Fig. 2, indicating the hydrodynamic relevance of sinuosity.

**Table 2:** Characteristics of the study sites.

Stream	Code	Latitude,	Bedrock	Drainage	Discharge	Sinuosity	Substratum characteristics
		longitude	geology		(m^3^ s^−1^)		
Moosach	M	48°23′33.90′′N 11°43′34.98′′E	calcareous	Danube	0.42	0.06	predominantly muddy
Wiesent	W	49°58′22.67′′N 11°11′29.40′′E	calcareous	Rhine	0.38	0.2	compacted coarse gravel
Guenz	G	48°16′16.38′′N 10°19′31.52′′E	calcareous	Danube	0.5	0.3	loose coarse gravel
Grosse Ohe	O	48°43′48.05′′N 13°15′14.37′′E	siliceous	Danube	0.61	0.6	sandy
Perlenbach	P	50°13′1.21′′N12°6′6.70′′E	siliceous	Elbe	0.5	0.7	loose fine gravel
Suedl. Regnitz	R	50°17′7.85′′N 11°59′34.16′′E	siliceous	Elbe	0.88	1.0	sandy

Characteristics of the investigated stream reaches: code, geographical position (latitude, longitude), associated bedrock geology (calcareous or siliceous), drainage system, mean annual discharge, sinuosity calculated for 1.5 km linear distance upstream of the investigated stream reach, and substratum characteristics.

Both models (Eqn 1 and Eqn 2) were calculated from the development data. The validation data (open circles in Fig. 5), which were not used to calculate the regressions, confirmed the models, as they did not show substantial deviation from the models.

## Discussion

To our knowledge, this is the first study which comprehensively quantified the spatial variability of a series of standard parameters which are used to characterize the physico-chemical substratum conditions in the hyporheic zone. The results indicate that a prediction of the spatial variability of a stream reach is possible prior to sampling. To this end, easily determinable variables (sinuosity and macrophyte cover) which drive spatial variability are required to deduce recommendations for sampling. Since driver properties and the spatial variability vary between stream reaches within one stream and between streams, sampling designs have to be adapted to the unique local situation of the spatial variability of each stream reach. It is likely that the suggested geostatistical approach applied herein will also be useful for determining sampling strategies in other habitat types and ecosystems as well.

### Drivers of Spatial Variability

The spatial variability, as represented by patch contrast and patch size, is driven by biotic and abiotic factors. In this study patch contrasts (and thus also the variance) of hyporheic parameters increased with the macrophyte cover. This is in line with results by Schulz and Guecker [21] and Svendsen and Kronvang [22], who found that submerged macrophytes have a considerable effect on the spatial variability. Submerged macrophytes reduce the current speed above the substratum (within the assemblages of submerged macrophytes) and thus enhance nutrient and sediment retention. In particular, submerged macrophytes should favor deposition of fines and slow down the exchange of interstitial pore water with the free-flowing water body. This explains our result that samples within macrophyte assemblages had significantly higher specific conductance, but significantly less dissolved oxygen and lower pH values compared to samples outside macrophyte assemblages. The bacterial decomposition of seceded plant material will additionally enhance these effects [23]. It is remarkable that the highly significant correlation between macropyhte cover and patch contrast (Eqn 1) explains more than 90% of the variation of patch contrast data, irrespective of individual macrophyte species, growth types or growth stages. The parameter macrophyte cover can easily be measured and is thus a sufficient indicator for an *a priori* estimation of the stream reach specific patch contrast. Patch contrasts and macrophyte cover cannot increase to an infinite degree; as the patch contrast is based on a co-occurrence of different patch types, it is to some extent speculative up to which degree of macrophyte cover Eqn1 holds true. However, it is unlikely that a stream will ever be completely covered by macrophytes, and most natural stream habitat types should lie within the range of macrophyte cover for which the equation was developed.

The definition of a patch is the maximum diameter of an area differing more from its surroundings than inside. Keller and Melhorn [24] described that patch sizes increase with increasing stream width. In contrast, the stream width was no driver for patch size in this study, which is likely based on the fact that the stream width variation between investigated stream reaches was minor in this study. Remarkably, our study revealed a second driver for patch sizes. For streams similar in width (all <10 m), in mean annual discharge (all <1 m^3 ^s^−1^) and current speeds (mean 0.25 m s^−1^), we found that the patch sizes of hyporheic parameters decreased with increasing sinuosity. Patch sizes were smaller for stronger meandering streams and larger for minor meandering streams. Since our analysis did not reveal nested variograms, patch-in-patch structures could not be shown for the investigated scales. The high hyporheic patchiness revealed in this study (i.e. maximum patches sizes of 2 m in stronger meandering rivers) implies that the physico-chemical characteristics of the substratum change frequently in 5 cm substratum depth. Macroscopically, this is evident by a close succession of different patch types, e.g. mud pack and gravel runs, which are characterized by different substratum compositions affecting the substratum permeability and thus the exchange with the free-flowing water. Whether similar rules for the patch patterns also apply in other depths than in 5 cm still needs to be analyzed. However, since the drivers for the pattern suggest a connection with the sedimentation process, we may expect similar properties of the pattern also at greater depths as long as these sediments were deposited under similar flow regimes as those in 5 cm depths. This does not imply that different depths are similar in absolute terms but they can exhibit large contrasts (e.g. [6]). It even does not imply that the positionings of the patterns in different depths are identical.

In accordance with the beads-on-a-string concept [25] there can be less autocorrelation (strong gradients) between two adjacent sites that correspond to “two beads”, which receive exfiltration water from different source regions, compared to two subsurface sites that are connected within the hyporheic corridor without additional inflow. Such “beads” probably involve zonation of the hyporheos [25]. Although they may occur throughout the hyporheic corridor at different spatial scales they are presumably too large to be captured by our sampling strategy if they do not form potholes that were not evident in our rivers.

According to our model, hyporheic patch sizes of 2 m can be expected in stronger meandering rivers (sinuosity: 0.6). In contrast, patch sizes of 4 m can be expected in more channel-like rivers (sinuosity: 0.1). Although there is some difference between both geochemistry units regarding sinuosity and thus also patch size, the relation between sinuosity and patch size also holds within one stream (see validation) and within one geochemistry unit. Hence, we are confident, that sinuosity and not the geochemistry unit is the main driver for patch size. In line with Lorenz *et al*. [26], we assume that higher hydrodynamic variability (which is induced by stronger meandering) leads to a more heterogeneous sorting of stream substratum and thus to smaller patch sizes. This is supported by our result that Reynolds numbers increased significantly with increasing sinuosity. Increasing turbulences, as indicated by increasing Reynolds numbers, should cause decreasing eddy sizes (swirls of turbulent water) and thus reduce patch sizes. This is also supported by findings of Davis and Barmuta [27], who associated high patchiness with high Reynolds numbers. Additional studies that prove the link between hydrodynamics, in particular the Reynolds number, and ecological processes are given by [28], [29], [30]. Even though hydrodynamic parameters like Reynolds numbers better describe the physical influences on patch sizes, sinuosity is easier to apply for planning of a sampling design. It does not depend on *a priori* measurements of variable flow velocities and hydraulic radii but it can easily be derived from readily available aerial photos. As evident from the correlation values between sinuosity values of different upstream section lengths and patch sizes (see results) a length of 1 to 1.5 km upstream of an intended sampling site is ideal for this purpose. Shorter distances may not characterize the degree of flow turbulence, induced by meandering, while longer distances may no longer be relevant for the sampling site. This supports the assumption that relatively small areas (in the scale of pool/riffle and reach) in stream ecosystems are influenced by progresses of landscape scales upstream (up to 1.5 km) of the investigated reach. As a consequence, successful stream restoration implies that improvements of even small stream reaches need to consider the catchment scale.

### Recommendations for Habitat Assessments

Basically, there are three cases regarding the investigation of a stream reach:

- The first is to infer mean and/or total of a population (including physico-chemical parameters) in a given section. A statistical test on significant differences between means (e.g. ANOVA) is not intended. In contrast, investigation of co-variance between means of different sections that are not autocorrelated may be intended. For this, a design-unbiased sampling (e.g. random sampling) is adequate even when applied to populations that exhibit spatial autocorrelation [31]. The estimator might not be the minimum variance unbiased estimator compared to an estimator from model-based sampling (which may be based on geostatistics), but there is not necessarily a bias in the point estimates [32]. Some designs explicitly make use of spatial autocorrelation. Adaptive cluster sampling designs, along with many designs derived from this basic design, all seek to exploit clustering that is inherent to a population in order to improve estimates of rare characteristics or events. Again, the estimators have been derived to provide unbiased estimates of mean and total (see [32] for a detailed discussion). Such unbiased estimators (e.g. mean) are unbiased for the sampled data, but do not compulsorily represent the river section adequately. If the estimator should represent a wider area, e.g. the reach (10^1^ m), care needs to be taken by defining the area to be sampled. The sampled area should be wide enough to capture the full range of values for the measured parameters and thus be representative, e.g. if we want to compare the hyporhoes with the fish population in a reach. Predicting patch sizes according to Eqn 2 may help to select an adequate area for this purpose.
- The second case is to infer means and/or totals from different sections and to compare them statistically (e.g. ANOVA) or to investigate the co-variance (e.g. regression) between the measurements of point samples. In this case, spatial autocorrelation needs to be addressed, since data obtained within a spatial pattern are not independent, which is a critical assumption in statistical standard tests [31]. To achieve this, there are two possibilities: avoid or correct spatial autocorrelation. In the first case, samples that are not autocorrelated are mandatory to avoid pseudoreplication (i.e. underestimation of variance) [33]. To this end, sampling distances should be larger than the maximum patch sizes. Following Eqn 2, this requires sampling distances of 2 m in stronger meandering streams (sinuosity: 0.6) and sampling distances of 4 m in minor meandering streams (sinuosity: 0.1). These suggestions are meant to avoid the introduction of a bias caused by sampling of spatially autocorrelated data and are thus valid for all possible sampling designs (random sampling or any kind of systematic sampling design). In the second case sampling distances smaller than the patch size can be chosen. Hence basing the sampling distances on sinuosity is recommended again. At distances of one fourth of the expected maximum patch size, most patches and gradients within should be covered. Following Eqn 2, such an approach requires sampling distances of 0.5 m in stronger meandering streams (sinuosity: 0.6) and sampling distances of 1 m in minor meandering streams (sinuosity: 0.1). Whilst this approach provides more detailed spatial resolution, effects of autocorrelation need to corrected, e.g. by including into the error variance of the statistical model (for a brief methodological overview see [34]) or by adapting the degrees of freedom [35]. Consequently, if autocorrelation is to be included in a statistical model, at least 100 but better around 200 samples are needed for a precise estimation of the autocorrelation structure [36].
- If a sampling procedure is destructive, but measurements from the same location are needed, e.g. analysis of co-variance between *in situ* redox-potential measurements and *ex situ* oxygen concentration measurements, we need to obtain the second sample from neighboring points. Further, a geostatistical analysis is mandatory, e.g. space interpolation (kriging). The same is true if we are interested in small-scaled pattern analysis of a property itself. Since Kerry and Oliver [18] suggest sampling distances less than half of the local patch size, the same distances as in (ii) can be recommended.

The number of necessary samples increases with required accuracy. The accuracy may be quantified as confidence interval of the mean, which is for normal distributed data given by:


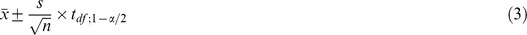


where 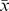 is the mean, *s* the standard deviation, *n* the number of measurements, *t* the tabulated t value depending on n and the desired probability of error α and *df* are the degrees of freedom which can be adapted according to the degree of spatial autocorrelation [35], [37]. With increasing macrophyte cover and patch contrast, sample numbers need to be increased.

There was no consistent difference between the two sampling dates in patch contrasts and patch sizes in all stream reaches for all parameters of a stream reach, despite of the differences in absolute values. This suggests that the same sampling design may be used throughout the year (excluding influential hyporheic events such as flooding). This is also supported by the fact that sinuosity does not change over the year and at least during the growing season, no change in the macrophyte cover could be observed. In contrast, the seasonal timing of sampling can be important for quality assessments, especially when limiting factors (e.g. a minimum discharge) are part of habitat functionality or suitability for certain species. Typically, low-flow conditions during summer and fall coincide with worst-case conditions of low oxygen supply in the hyporheic zone, which can be crucial for the survival of freshwater species [6]. It remains to be tested, whether the approach can also be applied to other stream types in different environmental settings.

### Conclusions

Spatial variability, as represented by patch contrast and patch size, was driven by biotic (submerged macrophytes) and abiotic (sinuosity) factors. While the patch contrast of hyporheic parameters increased with macrophyte cover at the investigated stream reach itself, patch sizes decreased with increasing sinuosity of the stream course upstream of the investigated reach. These relationships were remarkably similar among different physico-chemical parameters of the hyporheic zone and among different times in the year. For unbiased assessment of the hyporheic parameters, we recommend sampling distances be inversely related to sinuosity 1.5 km upstream, while relating the total number of samples to submerged macrophyte cover of the stream reach.

## Materials and Methods

### Stream Reaches and Sampling Procedure

Despite the fact that no specific permits were required for the described field studies, water authorities were informed about the sampling procedure and dates in the specific streams.

Six streams, situated in the three major drainage systems of Central Europe (Elbe, Danube, Rhine), were studied (Fig. 1, Table 1, 2). These were Moosach (M), Wiesent (W), Guenz (G), Grosse Ohe (O), Perlenbach (P) and Suedliche Regnitz (R). They represent relatively small (width <7 m, depth <1 m and mean annual discharge <1 m^3^ s^−1^) streams in a relatively cool, humid, sub-oceanic climate. In each stream, one stream reach (approx. 15 m long) was chosen to obtain the maximum between – reach variance in properties that likely govern hyporheic heterogeneity. These properties included bedrock geology (calcareous and siliceous), plan view morphology and presence of macrophytes. A summary of statistically significant differences between stream reaches is shown in Table 1. To include temporal variation, sampling was predominantly carried out on two sampling dates, in spring (June 2010) and in fall (September 2010). These data were used to develop statistical models for the spatial variability (development data). Two additional subsamples were taken in August 2011 in stream M at distinct stream reaches for model validation (validation data).

Samples were collected using a nested sampling design (Fig. 1); this is particularly recommended if the scale of variation is unknown [38], as different degrees of variance may be observed depending on the scale of sampling [39]. Since a nested sampling design allows to sample at multiple scales across the habitat, it can account for multiple degrees of variation. Thus, we nested two rectangular sampling grids to comprise two spatial scales, pool/riffle (10^0^ m) and reach (10^1^ m, as defined by [40]). At the pool/riffle scale, 49 samples per m^2^ were collected in a 17×17 cm grid. They were nested in the 12 to 15 m long, by 3 to 6 m wide reach scale sampling areas (45–90 m^2^). Stream reaches were sampled in a 1×1 m grid (113–161 samples). Reach length and width varied because they were adapted to the local situation of the stream reach (Fig. 2). The location of both, reach and pool/riffle scale grid remained constant between sampling dates.

At each sampling site, a series of standard parameters were measured to characterize the flow conditions in the water body and the physico-chemical conditions in the hyporheic zone. Flow conditions were characterized by 1) water depth, measured with a graduated rod (±1 cm) and 2) current speeds 5 cm below water surface and 5 cm above substratum, measured with a HFA flow measuring instrument (±0.01 ms^−1^, Höntzsch Instrumente, Waiblingen, Germany). Current speed was measured for 10 s and the mean value was derived. To characterize the physico-chemical substratum conditions of the hyporheic zone, interstitial pore water from 5 cm depth was extracted using an aluminum pipe (internal diameter 6 mm) inserted into the substratum. A syringe (volume 100 mL, Braun, Melsungen, Germany) attached to a plastic hose (internal diameter 8 mm) was connected to the aluminum pipe; the syringe was used to create negative pressure. Extracted water was transferred into Falcon tubes (ROTH, Karlsruhe, Germany) to measure pH, specific conductance (corrected to 25°C) and dissolved oxygen using handheld Multi-3430 G equipment (WTW, Weilheim, Germany). To enable correction for a diurnal trend, the time of each measurement was recorded. To verify that a diurnal trend was not misinterpreted as a spatial gradient along the sampled stream reach, samples of the free-flowing water were collected at one position and measured (every 30 min to accomplish approx. 12 samples) in the same way as the interstitial pore water. Additionally, the presence or absence of submerged macrophytes at each sampling site was recorded (Fig. 2).

The plan view morphology of the stream courses upstream of the sampled stream reaches (Fig. 1; middle panel) were characterized regarding the degree of meandering as indicated by the sinuosity. The sinuosity was calculated as the ratio of flow length to linear distance minus one. A sinuosity of 0.3 thus indicates that the flow path is 30% longer than a hypothetical linear distance between two points. Since the sinuosity of a stream may change, depending on the linear distance taken into account, we calculated and compared sinuosity for 0.2 km, 0.5 km, 1 km, 1.5 km and 2 km of linear distances. Data were available from the state surveying office (Landesvermessungsamt Bayern, www.geodaten.bayern.de/BayernViewer2.0/).

### Data Analysis

#### Step A: Correcting temporal trends

In order to quantify spatial variability, additional temporal variation caused by diurnal trends during the day-long sampling (e.g. changing dissolved oxygen values caused by changing algal photosynthetic activity) had to be separated from the spatial variability. Such diurnal trends are typical for stream ecosystems [41] and lead also to an apparent temporal autocorrelation which disguises the spatial autocorrelation. To this end, we examined the data for a temporal trend, using linear regression. Therefore, linear regression was calculated between time of sampling (independent variable) and corresponding values of the measured parameters (dependent variable). A temporal trend was assumed if the slope of the regression was significantly different from zero, and could be confirmed by the data of the free-flowing water. Consequently, the trend was removed by subtracting the slope of linear regression model from the data.

#### Step B: Characterizing stream reaches

The stream reaches were characterized according to flow conditions in the water body and to physico-chemical conditions in the hyporheic zone. Significant differences in the mean values of these parameters were investigated between stream reaches. To avoid pseudoreplication, we used an ANOVA based on generalized least squares, which allowed to simultaneously quantify and to correct for spatial autocorrelation. Computation was done in R 2.11.1 [42] with the auxiliary package nlme [43].

#### Step C: Determining patch contrast and patch size

The spatial autocorrelation was analyzed by calculating variograms for each parameter of each stream reach, and each sample date. The variograms allow investigating the elements of spatial variability [44], [45], which are (i) the sill (subsequently denoted *patch contrast*, as indicated by the maximum of the model) representing the patch to patch variance and (ii) the range (subsequently denoted *patch size,* as indicated by the distance at which the patch contrast is reached), representing the extent of spatial autocorrelation synonymously with spatial dependence (Fig. 3). To retrieve patch contrast and patch size, a spherical variogram model was fitted to the variogram data, since the transition to the sill (the spatial dependence) often follows the shape of a sphere (see Fig. 3) [19]. Patch contrast and patch size do not describe an individual patch but integrate all patches within the sampled stream reach. For further details of geostatistics see [46] and references therein. The variograms were calculated in R 2.11.1 with the auxiliary package gstat [47]. Goodness of fit was evaluated as r^2^ and was always greater than 0.85.

#### Step D: Normalizing patch contrasts

Since semivariances and hence also their maximum, the patch contrasts, of different parameters had different units (e.g. µS cm^−1^ for specific conductance and mg L^−1^ for dissolved oxygen), they had to be normalized. Normalization was done by dividing the individual patch contrasts of each parameter by their squared global mean. This yielded dimensionless patch contrasts that could be quantitatively compared between parameters.

#### Step E: Determining differences between stream reaches

Patch size and normalized patch contrast were tested on equal means with ANOVA. To control for type one error rate in case of significance and categorical factors with more than two levels, Fisher’s LSD post hoc test was applied. Significance levels of p<0.05, p<0.01 and p<0.001 were used. Data and residuals were checked for normality and homogeneity of variance. The ANOVA was computed in STATISTICA 8.0 [48].

#### Step F: Correlating patch contrast and patch size to drivers

The ANOVA showed that the patch size and the dimensionless patch contrast of all hyporheic parameters differed between stream reaches (Fig. 3, compare R and M). Regressions with stream properties that were assumed as potential drivers of the spatial variability were then calculated. To accurately fit the data, different statistical models were calculated for each potential driver and compared: These were linear model, power function and saturation model. In case of significance, the AICc values of these models were compared [49]. Finally, we present the model with the lowest AICc for the correlation with patch contrast and patch size, since these are supposed to be the optimum models among all other significant models. The obtained models should allow the prediction of the autocorrelation, based on the driver properties (within the defined range of streams).

#### Step G: Validation

For validation of the models (Fig. 5), two additional stream reaches in stream M were sampled and analyzed. These two reaches differed in their driver properties (sinuosity and macrophyte cover) from those previously sampled in this stream. Sampling design and statistical treatment for the validation data set was identical to the development data set but included a reduced set of parameters (specific conductance and pH) limited to the hyporheic zone. The stream M was selected for the validation study because it constituted one extreme reach in terms of sinuosity and macrophyte cover compared to that of the development data set. The question arose whether less extreme reaches within the same stream would follow our models or whether they would deviate (showing similar values as the extreme reach in M), thus indicating that the relations would not be general but stream-specific. The data set was reduced to hyporheic parameters, because they are characterized by a high sensitivity to the driver properties. Within the hyporheic parameters, dissolved oxygen measurement (which was strongly correlated to pH) was excluded, because we found that the models should hold for any of the tested physico-chemical parameter in the hyporheic zone. Validations were carried out in a different year and month (August 2011) to test the independence of the relations from the annually and seasonally varying conditions.

#### Step H: Deducing recommendations for sampling design

Recommendations for sampling designs can be predicted through the development of relational models (Eqn 1–3) resulting from the above analysis.
